# Experimental and Inverse Analysis of Natural Cork Material with Anisotropic Nonlinear Viscoelastic Behavior

**DOI:** 10.3390/polym18212722

**Published:** 2026-11-02

**Authors:** Cylia Yahiaoui, Younès Saadallah, Reza Afshar, Kristofer Gamstedt, B. Emek Abali

**Affiliations:** 1 Laboratoire Mécatronique (LMT), Faculty of Sciences and Technology, University of Jijel, Jijel 18000, Algeria; cylia.yahiaoui@univ-jijel.dz; 2 Laboratoire de Génie Mécanique-Jijel, University of Jijel, Jijel 18000, Algeria; y.saadallah@univ-jijel.dz; 3 Department of Materials Science and Engineering, Division of Applied Mechanics, Uppsala University, Box 524, 751 20 Uppsala, Sweden; reza.afshar@angstrom.uu.se (R.A.); kristofer.gamstedt@angstrom.uu.se (K.G.)

**Keywords:** anisotropy, creep recovery, loading–unloading cycles, Mullins effect, hyperelasticity, inverse analysis

## Abstract

Due to its cellular structure, cork from Quercus suber has significant deformability, showing magnificent recovery after being compressed by 50%. Its mechanical characteristics show dependency on the direction of the external load (anisotropy), making it difficult to estimate recoverable large displacements. Here, the mechanical behavior of cork is investigated under loading–unloading cycles (1–15 MPa) in the radial and non-radial directions. Cork demonstrates higher stiffness and higher residual strain in the radial direction, compared to the softer, buckling-dominated behavior in the non-radial direction. Both directions feature significant cycle-by-cycle softening, suggesting a Mullins-type (recoverable) damage mechanism, with residual strain increasing in both directions. According to the data obtained six months after testing, dimensional recovery demonstrates ongoing recovery with varying percentages depending on the direction (92% against 88% of the original height after six months). It supports the theory of softening from cycle to cycle because the damage is primarily pseudo-elastic and therefore recoverable rather than irreversible. A transversely isotropic hyperelastic constitutive model is enriched with Mullins-type (pseudo-elastic) damage and viscoelasticity. The three-dimensional model is based on engineering applications; all parameters are obtained through inverse analysis of loading–unloading tests. Consequently, this model accounts for the anisotropic behavior and increasing recoverable damage of cork, allowing better prediction of its mechanical performance.

## 1. Introduction

Natural materials underpin a wide range of industrial sectors, offering viable alternatives to synthetic products with substantially reduced ecological impacts. In this context, cork stands out as a fully renewable, carbon-negative biomaterial obtained from the bark of *Quercus suber* L. [1]. The extraction of cork does not damage the tree because the bark regenerates after harvest; this is carried out by periodically removing the outer layer of the bark of the tree [2]. The forests of cork oak are found in a large region spanning southern Europe to north Africa. Recent estimates put the area of cork oak forests at approximately 2.1 million hectares, with reported cork extraction ranging from about 200,000 tons to 280,000 tons [3,4]. Therefore, cork is frequently mentioned as a paradigmatic resource within circular economy frameworks.

As a natural material, cork has exceptional mechanical and functional performance that is governed by its distinctive closed-cell honeycomb microstructure and its chemical composition, which is dominated by suberin (23.1–54.2%), lignin (17.1–36.4%), and polysaccharides (around 20%) [5,6,7]. This composition imparts remarkable compressibility, enabling strains approaching 80% with nearly complete shape recovery; such a level of reversible deformation is rarely attainable with synthetic foams [8,9]. In comparison with other types of biomass materials such as woods, cork possesses a unique pair of properties, including low density, strong moisture resistance, and antimicrobial properties [10,11], which make cork a perfect option for use as stuffing or core of bio-based composites. Effected by this unique set of properties, cork has attracted increasing interest for advanced engineering applications where repeated large deformation, energy absorption, and long-term durability are required under service conditions [1,5,12,13]. However, despite an increase in interest, cork-based components are still mainly designed through trial and error and without using predictive modeling techniques since existing constitutive models of cork usually take little account of cork’s strong anisotropy, cyclic softening, or its long-term recoverability in conditions close to real-life ones. In order to facilitate efforts to make use of the potential of cork materials in load-carrying applications and as a constituent in bio-based composites, improved models and characterization methods are needed to predict the mechanical behavior under such service conditions.

Constitutive models for cork and similar cellular materials with a possibly multiscale architecture [14] have mainly focused on the interrelation between cellular structure and quasi-static compressive properties. Earlier studies, including the pioneering ones by Gibson, Easterling, and Ashby [15], demonstrated the relationship between the honeycomb cell pattern of cork and its compression behavior, thus laying the foundation for the future explanations of cork’s mechanical behavior. Rosa and Fortes [16] expanded the idea of how strain rate affects the characteristics of cork in compression and recovery, emphasizing the extent to which the deformation behavior is dependent on time. Since those early studies, further research has been carried out about the mechanical behavior of cork, and among others, we mention the compressive response of cork [8,17], its anisotropic and heterogeneous nature [18], and its nonlinear time-dependent properties [19]. Still, results mainly serve as empirical results and predictive constitutive models accounting for the behavior of cork characterized by nonlinearity, anisotropy, and time dependence are missing.

In general, hyperelastic strain energy formulations together with viscoelastic dissipation potentials have been extensively used to model the response of foams [20,21], elastomers, and other types of filled polymers under large deformations [22,23]. Cyclic loading may produce a pronounced stress softening phenomenon in some of the aforementioned materials [24,25]. The Mullins effect as a description of this phenomenon has been introduced by Mullins and Tobin in connection with filled rubbers as an application of the theory of pseudo-elastic damage [26]. Although it is often suggested that the Mullins effect occurs due to progressive damage in polymer materials, similar effects are recorded in the case of porous materials, due to the ongoing irreversible buckling of the structure and build-up of microstructural damage during multiple loadings [9,27]. Nevertheless, the modeling of Mullins-type behavior in cork has not been sufficiently covered, and at present, material modeling has been achieved mostly for agglomerated cork under dynamic loads, while the studies of natural cork are still very limited [28].

Specifically, cork has a cellular microstructure leading to anisotropic behavior at the macroscopic scale [18]. Anisotropy is caused by material structure differences. Similar observations have been noted for other structured material types, where the microstructure varies the response at the macroscopic length scale [29,30,31,32]. Cork is of special significance because the structure of the cork material indicates different deformation mechanisms and responses to compressive loading due to radial and non-radial loading. This anisotropy needs to be incorporated in modeling for the proper characterization of cork material.

Despite this long-standing technological relevance, the constitutive description of cork under complex mechanical and environmental loading remains comparatively underdeveloped. In particular, there is a need for robust material models capable of capturing the nonlinear, time-dependent response of cork under loading–unloading cycles and sustained loads, especially when these are coupled with environmental variables such as humidity. Under such conditions, time-dependent phenomena such as creep, stress relaxation, and hysteresis would strongly influence performance, yet they are not fully represented in many existing modeling frameworks.

The present work addresses this gap by developing a constitutive relation with a clear mathematical formulation suitable for three-dimensional numerical implementation. We show a relatively simple experimental campaign by combining loading and unloading with varying amplitude in the radial and non-radial directions to obtain the material parameters in the case of transverse isotropy. By using the knowledge that Poisson’s contraction is zero, we obtain the full set of material parameters only by means of uniaxial compression along radial and non-radial directions. The study aims to identify the mechanisms governing cork’s nonlinear and viscoelastic response and to establish predictive relationships between loading history and strain evolution. Herein, we use constant material parameters in the model and invite experimentalists to conduct tests for determining the dependency on humidity and temperature. This modeling approach is intended to support the design and optimization of cork-based components in demanding engineering applications, where accurate prediction of deformation and recovery under cyclic and sustained loads is critical.

This work models the aforementioned cork behavior as follows. Section 2 describes the material characteristics and methods of experimentation that lead to Section 3 demonstrating the results of the experiments. We construct a phenomenological constitutive (material) model in Section 3.3, involving anisotropy in elastic response, viscoelasticity, and stress softening. The discussion in Section 4 includes a comparison of experimental and modeling results from the perspective of microstructural behavior.

## 2. Materials and Methods

**Figure 1:**
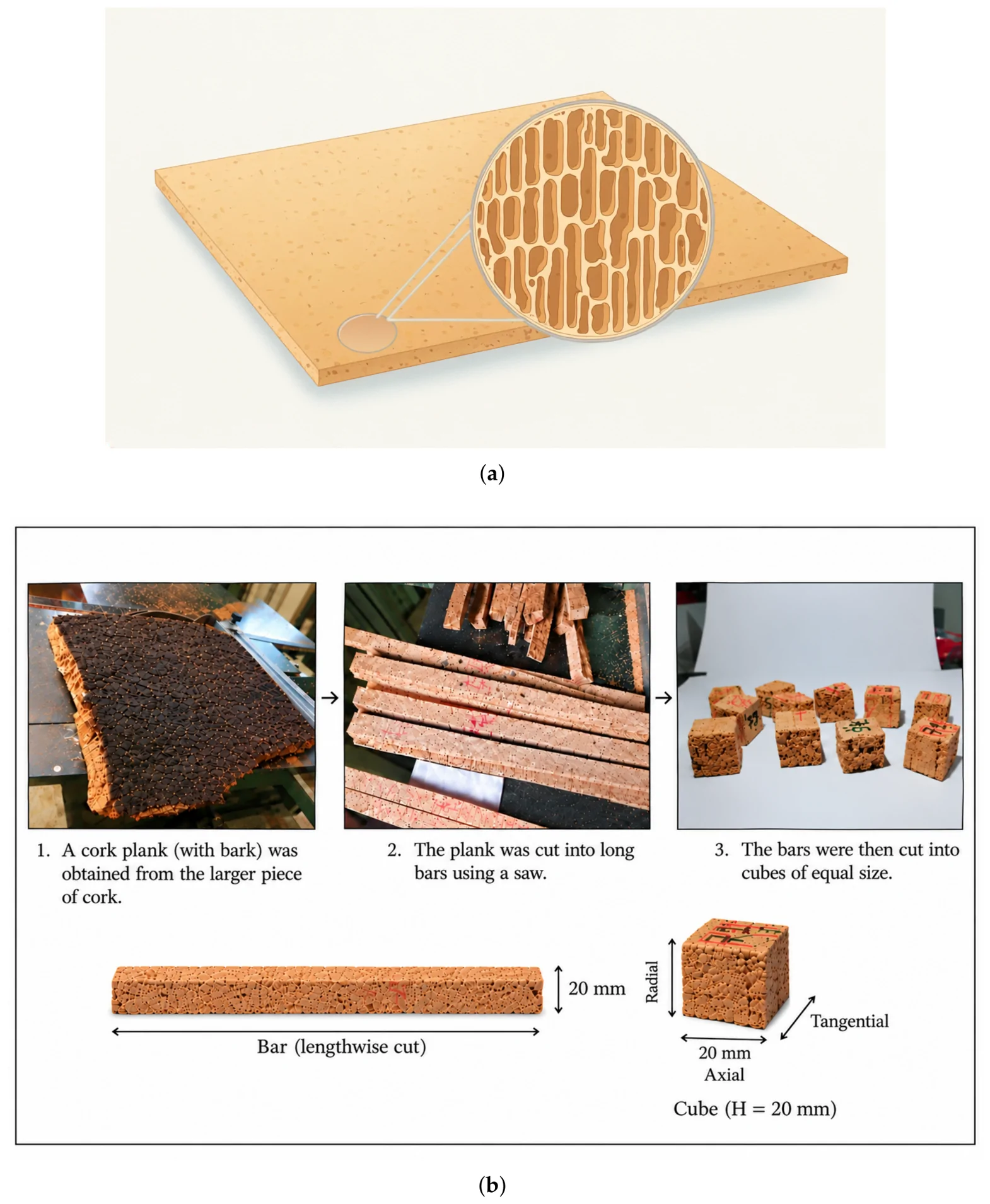
Cork anisotropy and sample preparation. (**a**) Illustration of the cork material with its cellular microstructure aligned in the radial direction (pith to bark), leading to a transversely isotropic response. The schematic plate is on the order of centimeters, while the zoomed-in cell-wall structure in the inset is at the micrometer scale. Picture credit to Adobe Firefly via the model of Gemini 3.1 (Nano Banana 2). (**b**) Procedure of sample preparation from raw cork to cubic samples. A cork bark section initially approximately 400 mm × 300 mm is cut into thin bars of 20 mm × 20 mm cross-section and the same length as the initial bark section, which are again cut into cubic samples of 20 mm × 20 mm × 20 mm on each side.

Natural and agglomerated cork materials may exhibit pronounced nonlinear compression- densification behavior, Mullins-type stress softening during reloading, and rate-dependent viscoelastic dissipation. In order to quantify these effects and their significance, we use a specific type of cork material and prepare cubic samples for compression tests. A simple but efficient protocol is developed to perform and fit all the material parameters in the constitutive model. We simplify the model by assuming that the anisotropy is captured by measuring the response in radial and non-radial directions. In other words, we simplify the transverse directions by assuming the axial and tangential directions are the same, and we hereafter refer to them as the non-radial direction, as illustrated in Figure 1. In this manner, a transversely isotropic model has been developed for the nonlinear elastic contribution by using deformation energy. Therefore, the model is ready to be employed in three-dimensional numerical calculations that are not the part of this work.

### 2.1. Sample Preparation

Samples were prepared from raw cork bark sections that had been extracted directly from the Jijel area in Algeria and obtained from the warehouses of Algeria’s National Cork Company in Jijel (Entreprise Nationale du liége). Each plank was examined and marked to distinguish its radial, tangential, and transversal (also called axial in the literature) directions, ensuring that the grain orientation had been understood. Longitudinal direction is parallel to the tree trunk’s axis while radial direction runs from the pith to the bark. Tangential direction is perpendicular to radial direction and follows the shape of the growth rings. Identification of the directions was carried out before sample preparation in order to maintain the uniformity of the sample position during machine testing. Firstly, the planks underwent boiling for 90 min at standard atmospheric pressure, which is an important step in the softening of the planks, making them suitable for further processing [33]. After boiling, they were air-dried for 48 h to remove excess moisture, allowing them to stabilize and reach ambient humidity levels. The moisture content of the specimens was not measured. After completing the drying process, the planks with a thickness of 25 to 30 mm along radial direction were cut into thin bars of 20 mm in width along the transversal (axial) direction such that the length of the slices are along the transversal direction. This step is depicted in Figure 1.

**Figure 2:**
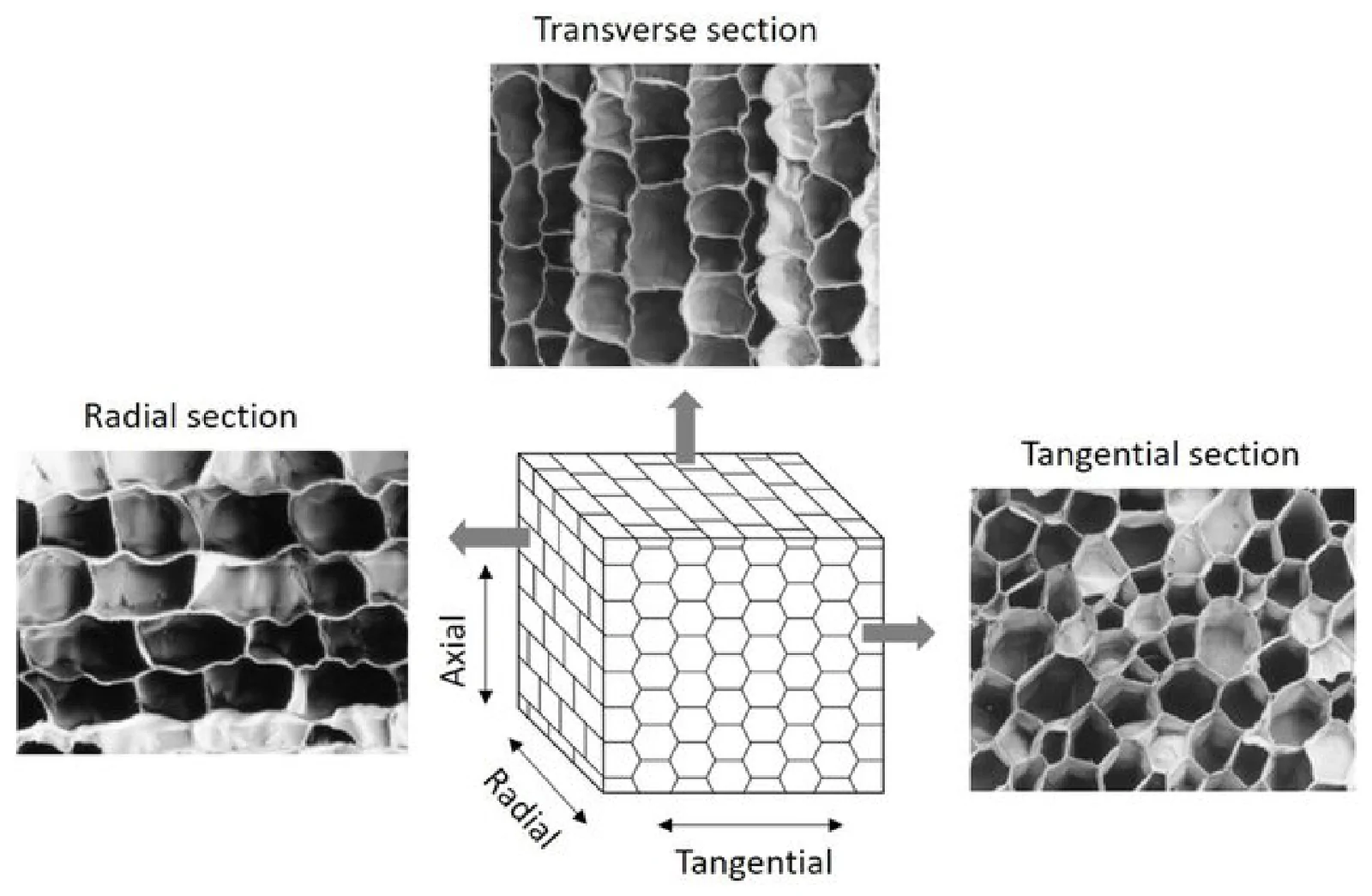
Schematic representation of the 3D cellular structure of cork and the structure of cork as observed by scanning electron microscopy in the three main sections: tangential section (perpendicular to the tree’s radial direction), transverse section (perpendicular to the tree’s axial direction), and radial section (the tree’s radial section). Adapted from [4], originally from [34], cross-sectional cell diameter is approximately 30–40 µm.

Figure 2 shows the cellular microstructure of cork, as well as the aforementioned radial, transversal, and tangential directions and their relationship to the orientation of cork cells. The aim of this illustration is to clarify the terminology used in the rest of this article, especially between radial and non-radial directions in mechanical tests.

In order to straighten the bars, the excessive thickness of each bar was removed radially, thus removing the bark side layer and a small portion of additional edge material until the thickness reached 20 mm. This approach ensured there were no irregularities and that the cross-section of every bar was 20 mm × 20 mm. After subsequent measurement to confirm the bar dimensions, the bars were cut into cubes with their edges in the tangential direction so that their original grain orientation was preserved. This procedure resulted in a number of cubes with dimensions 20 mm × 20 mm × 20 mm, with known material orientations, which were used for experiments. Following this preparation, cubes were weighed and measured in all three directions to accurately determine their mass density,

(1) $$\rho =\frac{m}{V}\;,$$

where the mass, *m*, and volume, *V*, of each specimen was used. The specimens had an average mass density of ρ=0.174±[0.037] $g/{\mathrm{cm}}^{3}$.

### 2.2. Mechanical Testing Procedures

**Figure 3:**
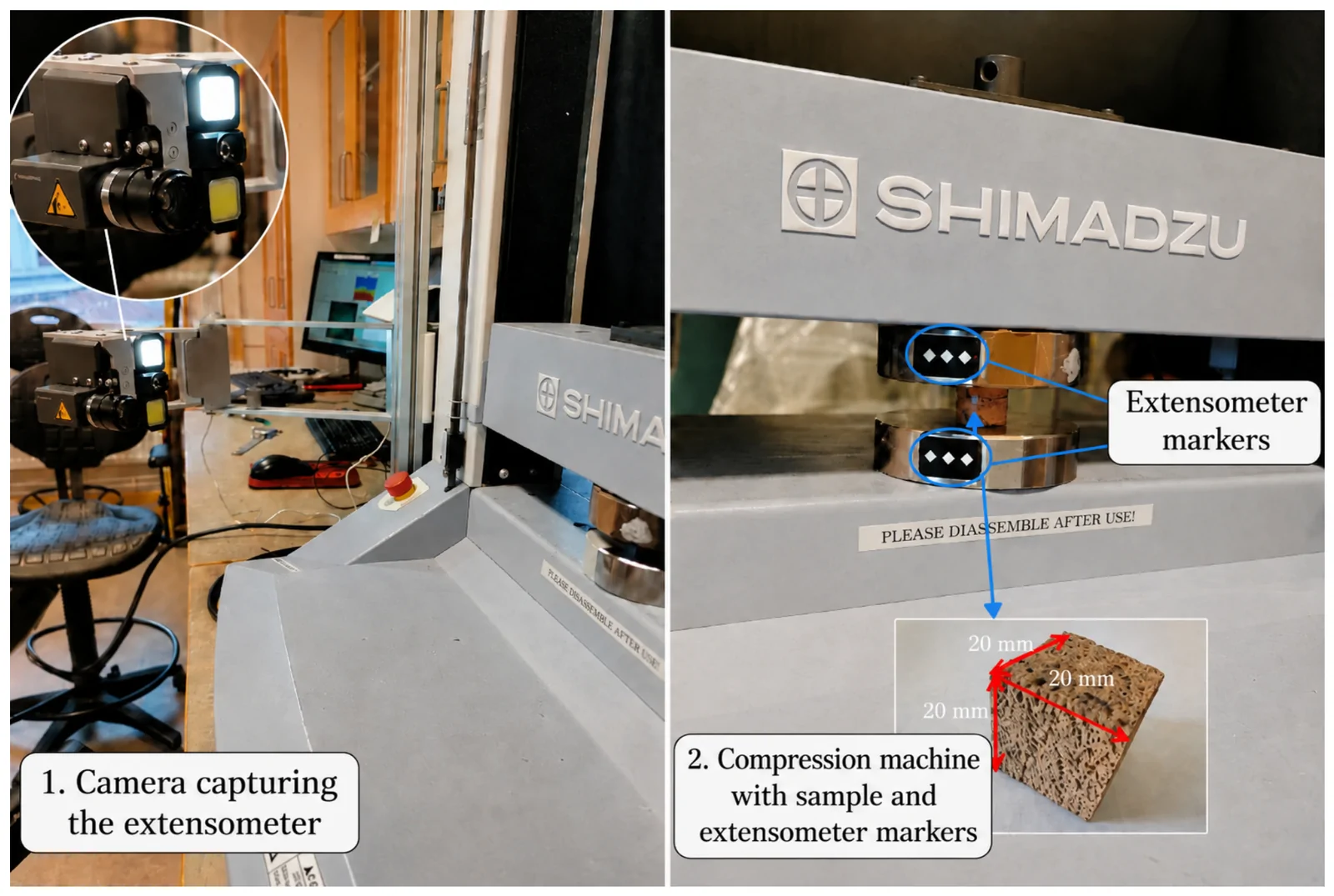
Experimental setup for uniaxial compression testing. (**1**) Non-contact video extensometer camera, used to optically track displacement during testing. (**2**) Shimadzu Autograph AGS-X compression machine with a cork cube mounted between the loading platens; the diamond-pattern targets on the upper and lower grips are the extensometer markers tracked by the camera in (**1**) to measure axial displacement (inset: representative 20 mm cork cube specimen).

Uniaxial compression tests were carried out using a Shimadzu Autograph AGS-X (Shimadzu Handelsgesellschaft GmbH, Korneuburg, Austria) universal testing machine, shown in Figure 3, equipped with a 10 kN load cell, with an accuracy of ±0.5% of the indicated test force.

The available speed range for the machine crosshead is 0.001–1000 mm/min, with a speed accuracy of ±1% and a maximum distance between the crosshead and the table of 1200 mm. Data acquisition and control of the machine were performed using TRAPEZIUM X software (version 1.5.1) provided by the manufacturer. The displacement was continuously recorded during the experiment with a non-contact extensometer (camera following marks), in a laboratory condition of controlled temperature of 21.5±0.5 °C and logged humidity of 40 ± 10% RH (Relative Humidity). As the extensometer, the camera was calibrated to track the positions of markers attached to the upper and lower crosshead to monitor the movement of the specimen between the jigs. We used a loading–unloading protocol to examine the viscous response with a varying amplitude to obtain the history-dependent mechanical behavior. The test samples underwent a series of loading–unloading cycles at seven progressively increasing stress levels of 1, 2, 3, 4, 5, 10, and 15 MPa. In other words, under a given strain rate, the compressive stress is increased until the stress level has been reached. After this point, by the same strain rate, an unloading has been started. This protocol is specific to the inverse analysis in what follows. The speed (compression rate) of loading–unloading was chosen so that each cycle is roughly the same duration during the first five cycles, that is 1 to 5 mm/min. Also, slower rates were used, 1 mm/min, for the last two cycles (10 and 15 MPa), allowing us to compare mechanical activity at several stress levels of the same loading cycle. In contrast, in the first five cycles, stress amplitude and loading rate doubled, tripled, and so on, in order to obtain the same loading time in the case of a linear viscoelastic material. We lack information on whether the material is linear viscoelastic a priori. By increasing the target stress with the loading rate, we ensure that each cycle lasts for a similar duration. We refer to Table 1 for the chosen protocol.

Both directions, radial and non-radial, were used to investigate the anisotropic nature of cork. In the radial cut, compressive stresses were applied along the cork cell axis, and in the non-radial cut, stresses were applied perpendicular to the cork cell axis, corresponding to the tangential direction for this study. Each test was repeated three times to evaluate its repeatability. Only the tangential direction was examined with regard to the non-radial direction. Within the cork mechanics literature, the transversal (axial) and tangential directions are similar relative to material properties; therefore, cork is usually modeled as a transversely isotropic material with the radial direction as the axis of symmetry [4,8,18,34]. As far as compression is concerned, cork is expected to behave in a similar manner regarding both axial and tangential directions and to display different properties when tested along the radial direction where its strength is known to be greater [35]. The evaluation of the tangential direction thus generates relevant information concerning the axial response. In this research, no tests were conducted to study the axial compressive response, and the equivalence of the two directions is reported in the literature [34].

After the completion of each loading–unloading cycle, the dimensional recovery of the cork material after compression was quantitatively evaluated. As a result of the very little side deformation seen during unidirectional pressure, it is fair to conclude that cork’s Poisson’s ratio is close to zero [15,36]. This observation justifies why dimensional restoration is believed to happen only in the direction of load application, and it was estimated based on the sample’s initial height. Specifically, the heights of the samples were recorded after unloading in regular rest periods of t=30 min, 1 h, 2 h, 24 h, 48 h, 72 h, 1 week, 2 weeks, 3 months, and 6 months. These values, h(t), were systematically compared with the initial heights, $h_{0}$, of the corresponding specimen in order to evaluate the percentage recovery of sample height as a function in time,

(2) $$\begin{array}{c} R\left(t\right)=\frac{h(t)}{h_{0}}\times 100\%\;. \end{array}$$

**Figure 4:**
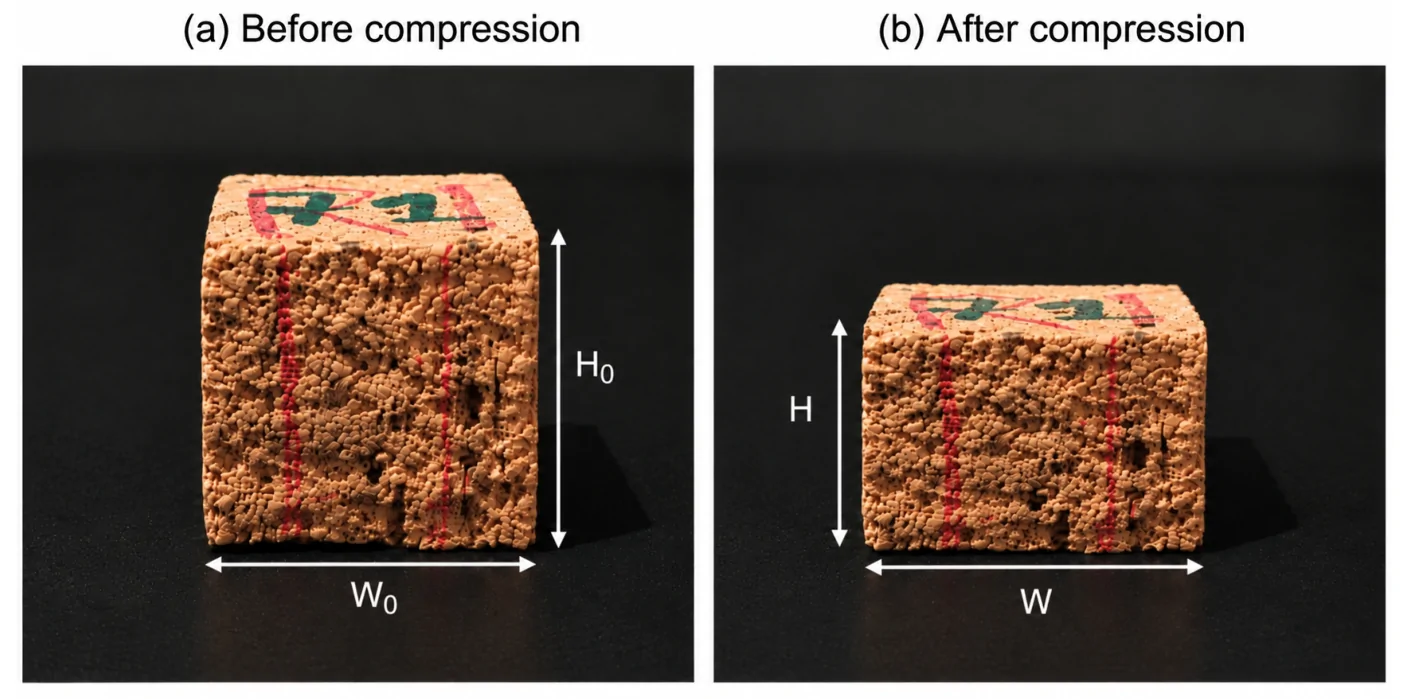
Representative cork specimen (20 mm cube side length) before and after compression, showing negligible lateral deformation and confirming that dimensional change occurs predominantly along the loading direction. $H_{0}$ and $W_{0}$ are the height and width of the sample before compression and *H* and *W* are the height and width of the sample after compression.

Axial compression of cubic specimens may cause friction at the interfaces between the specimen and the platens inhibiting lateral expansion in the vicinity of the surfaces. This constrain results in an uneven shape for the deformation and inevitable stress conditions that are not completely uniaxial [37]. It is anticipated, however, that cork will suffer little from this phenomenon as its near zero Poisson’s ratio (as demonstrated by the lack of significant lateral deformation in Figure 4) means that it will not undergo considerable lateral expansion anywhere and thus will not experience a barrel shape problem.

The data on loading–unloading and recovery presented above have been used as input for inverse finite element identification. A cork-specific constitutive model is developed and calibrated against the experimental results. As part of an effort to choose an adequate rheological model for cork, we have developed an inverse analysis procedure and tried linear rheological models such as Maxwell, Kelvin–Voigt, three-parameter solid, and a chain of these [38,39,40,41,42] and nonlinear elastic models [43,44,45,46]. We use nonlinear rheological material modeling by using energy [47], and we introduce assumptions of the anisotropy.

## 3. Results

In this section, the mechanical behavior of cork is studied experimentally, starting with the loading–unloading tests on different cuts in order to characterize its anisotropic and history-dependent response, which is followed by the analysis of dimensional recovery measurements.

### 3.1. Cyclic Loading

**Figure 5:**
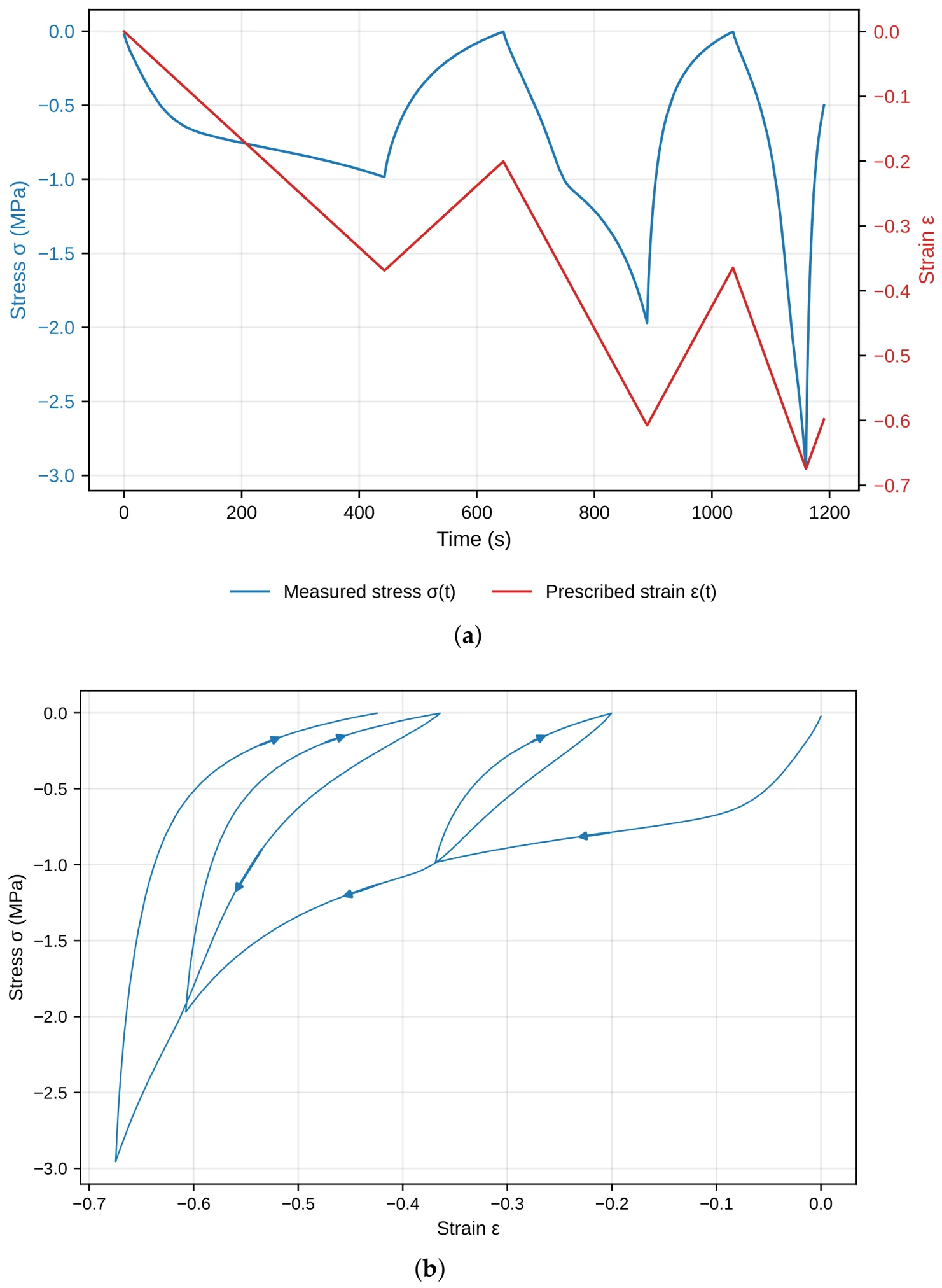
Loading–unloading response of cork in the non-radial direction for the first three cycles (1, 2, and 3 MPa). (**a**) Prescribed strain and its response as measured stress in time. (**b**) Corresponding stress–strain curve, showing the linear, plateau, and densification phases in cycles 1, 2, and 3, respectively, transient change is indicated by arrows.

In order to show the overall pattern of cork’s cyclic compressive reaction, we start with presenting the initial loading–unloading results before the full summary of results and responses in both directions. Figure 5 illustrates the initial cycles of loading and unloading conducted at three levels of compressive loads, namely, 1, 2, and 3 MPa along the non-radial direction with a loading rate of 1, 2, and 3 mm/min, respectively (see Table 1). The response is linear at first but changes quickly in slope and stiffens after about 50% of compression. The strain axis is defined in terms of measured values such that 0.7 means 70% of strain. The unloading curves are visibly nonlinear in all the cycles, and the shape (curvature) changes throughout loading and unloading cycles and the curves become progressively steeper through the cycles. Figure 5a depicts the loading process in terms of prescribed strain and measured stress with time, while Figure 5b shows the corresponding stress–strain curve that is also used for the inverse analysis.

**Figure 6:**
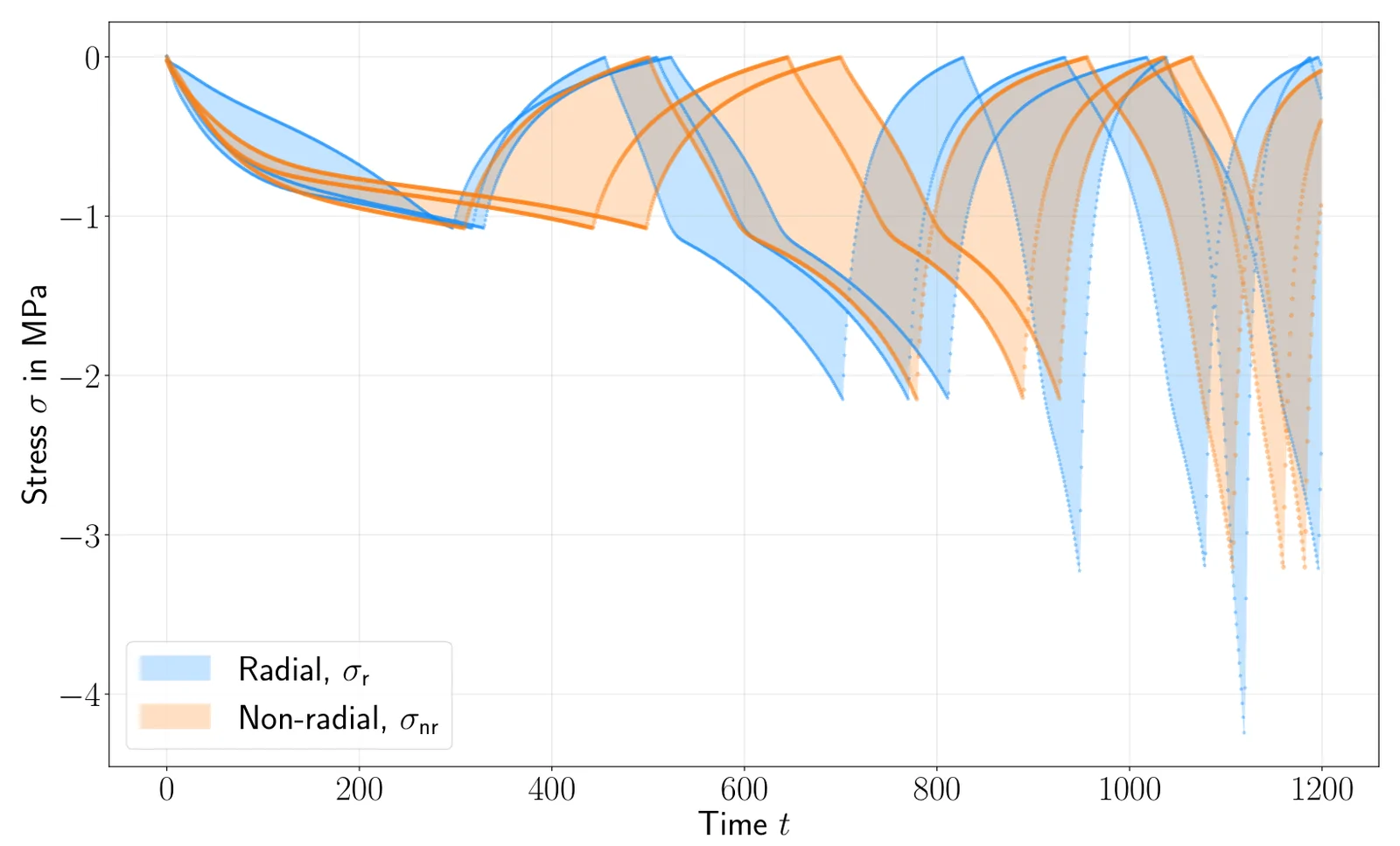
Stress responses of three specimens along the radial and three specimens along the non-radial directions are shown within 20 min of testing time for the protocol explained in Figure 5. The shaded areas indicate the dispersion between tests.

The stress–strain curves from the cyclic (loading–unloading) protocol with seven cycles as in Table 1 are presented in Figure 6. We emphasize the significant difference between radial and non-radial directions, although the material response shows similarities.

**Figure 7:**
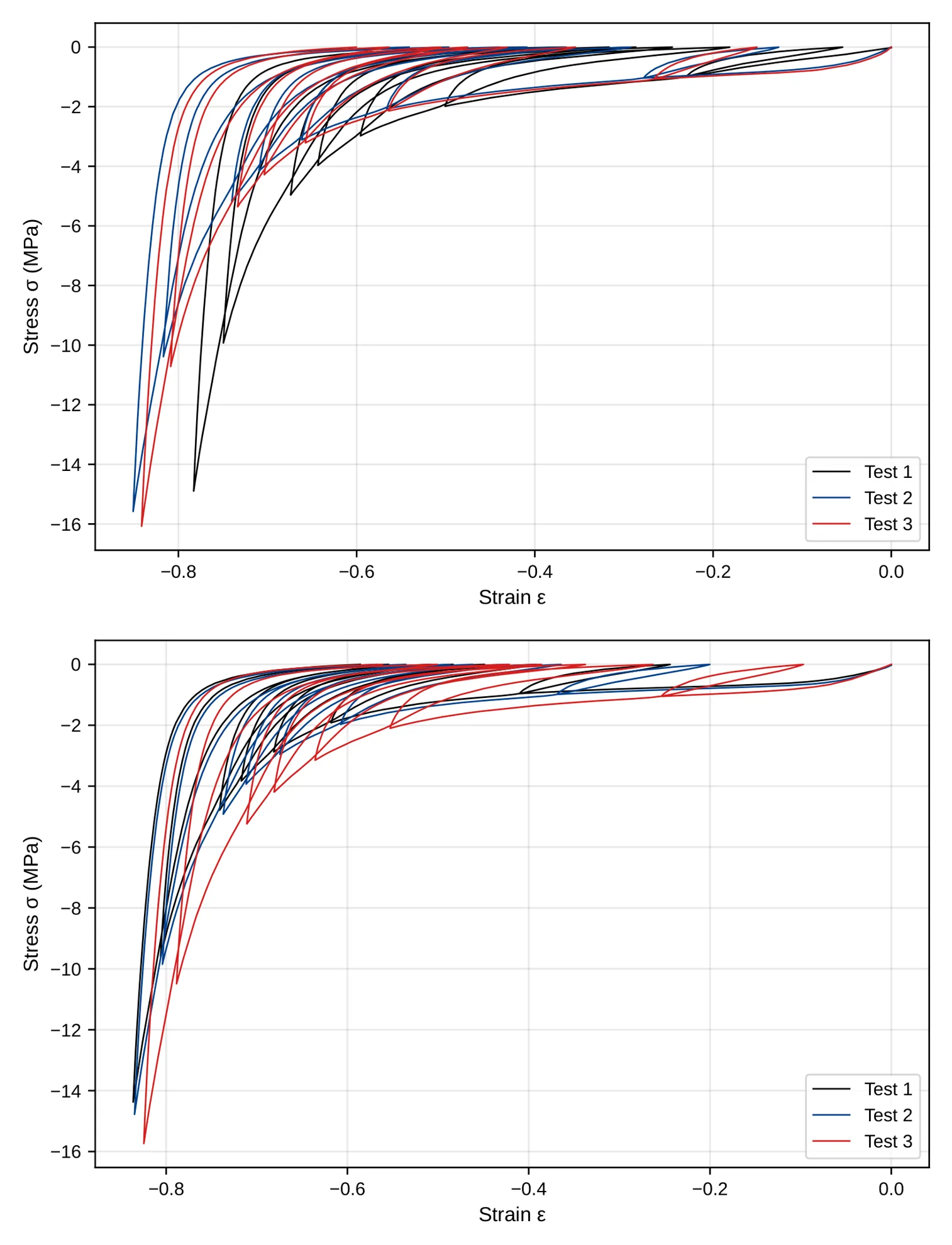
Repeatability of the loading–unloading response across three independent tests. (**Top**): Radial direction. (**Bottom**): Non-radial (tangential) direction.

The stress–strain curves have been obtained from the cyclic (loading–unloading) protocol with seven cycles as shown in Table 1. For each direction, three specimens are tested to quantify the repeatability. For the sake of clarity, we demonstrate only the first 20 min of experimental results in Figure 6 with a shaded area selecting at each time instant the minimum and maximum stress values. The tendency is repeatable; however, there is a sizable dispersion between results. This discrepancy seems to be caused by the first cyclic loading responding differently in a viscous manner such that the stress response is shifting in time. Specifically for one test in radial direction, the loading stage of the first cycle happens to be different. We may conclude that the first cyclic loading is setting the material and may be discarded as it is the usual approach in foams according to ISO 3386. Yet we lack a microscopic study providing the real reasoning for this behavior. Therefore, we keep the initial cycle as well in the inverse analysis and select one test in radial and non-radial cases as representatives. Both directions present a definite softening in successive cycles, with each new loading pass falling below the extrapolated response of the previous cycle. The same data are also shown in the stress–strain plots and in a total of six specimens for radial and non-radial directions, as shown in Figure 7. We summarize the variance in peak stress and strain in Table 2 in order to explain that the viscous behavior lacks the repeatability but elastic and stress softening may be evaluated as adequate.

The specific target concentration levels of stress have been chosen (1, 2, 3, 4, 5, 10, and 15 MPa), and at each test cycle, the compressive strain is taken to find out the relative spread of the stress and strain parameters over three specimens’ results by using

(3) $$\frac{\Delta X}{\bar{X}}=\frac{X_{\max}-X_{\min}}{|\bar{X}|}\times 100\%\;,$$

where *X* represents the peak stress or strain corresponding to certain target stress time instants and $X_{\max}$, $X_{\min}$, and $\bar{X}$ are the maximum, minimum, and average values of *X* for three specimens for the corresponding cycle. This process has been carried out individually for each target level of stress, resulting in obtaining one relative spread value per cycle per direction (Table 2). The relative stress spread, $\Delta \sigma /\bar{\sigma }$, is low at all stress levels and in both directions (Table 2). We emphasize that the experiment is conducted by controlling displacement, thus, strain and stress are measured. The relative strain spread, $\Delta \varepsilon /\bar{\varepsilon }$, exhibits a different behavior than the absolute strain spread. At the lowest stress level of 1 MPa, the relative strain spread is at the maximum for both directions. At this low stress level, the absolute strain values are also very small, so we guess that even small differences in the initial installation, leading to a differing contact condition between the samples, leads to significant differences in the initial cycle. Specifically, the very beginning of the loading curve manifests a seemingly softer response, resulting from the lack of complete or even initial contact of the sample with the loading plates prior to sufficient contact being achieved; minor specimen-to-specimen differences in this primary contact behavior, accordingly, lead to a higher stress response shift in time and strain variation. As the stress increases further, the material enters the stages of collapse and densification, where the strain behavior becomes more consistent among the samples, leading to a small relative strain spread. Across every level of stress, the relative strain spread is greater in the non-radial compared to the radial direction, with the distinction being most apparent at low stresses.

### 3.2. Recovery

The dimensional recovery of the cork samples are needed to understand if an elastic or plastic formulation is needed. This evaluation is simply carried out by measuring the sample height after the loading–unloading testing procedure. Figure 4 shows a comparison of the corresponding sample before and after the compression test, where the negligible lateral deformation indicates that the dimensional change occurs predominantly in the loading direction and in accordance with the zero Poisson’s ratio. There is no barreling effect, which normally exists in compressive tests of cubic samples due to friction-restricted side-volume expansion at the plates, which is in line with the zero Poisson’s ratio for cork and proves the absence of significant non-uniform deformation in the process.

**Figure 8:**
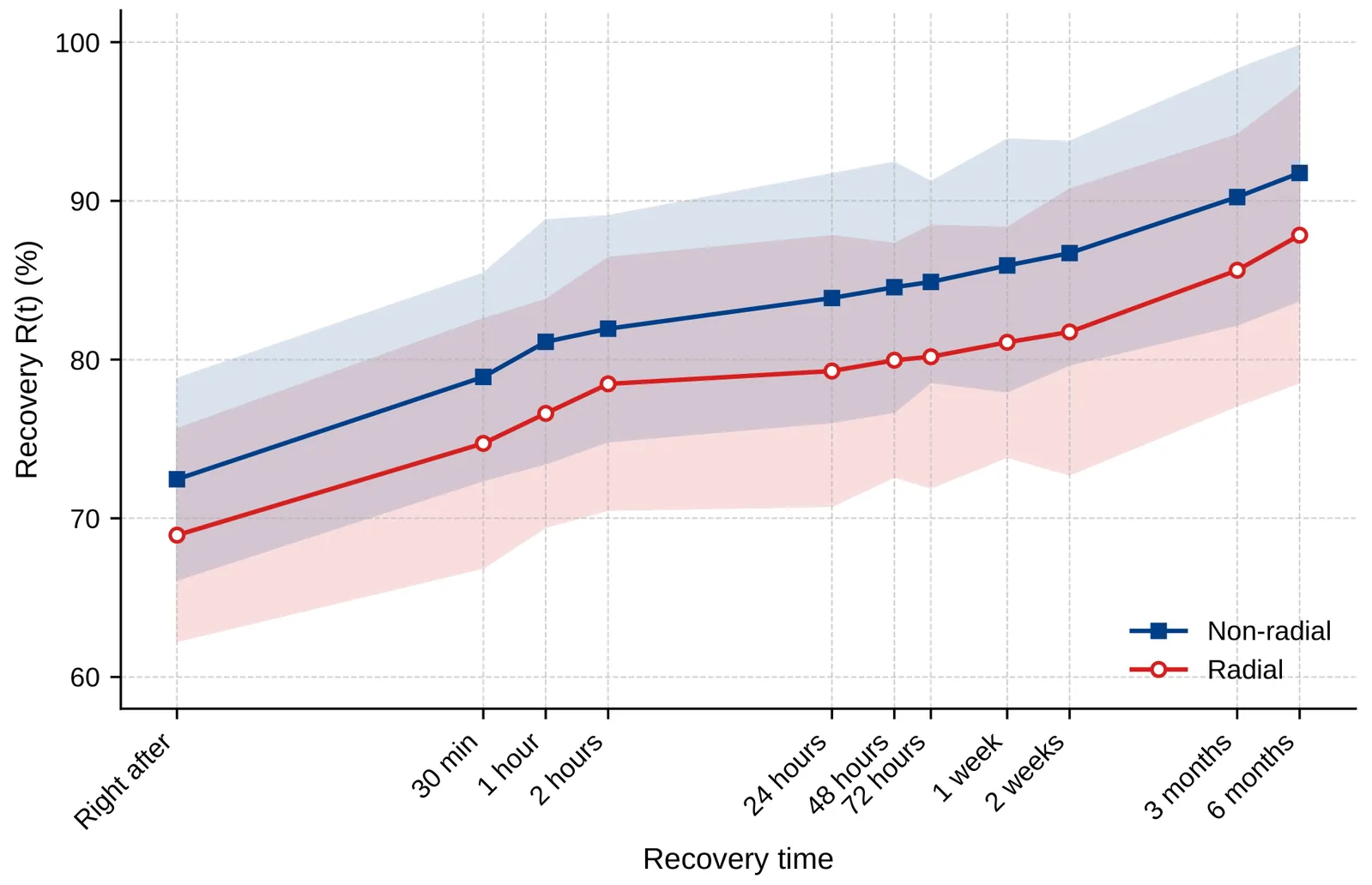
Height recovery R(t) of cork cubes as a function of resting time after unloading, on a logarithmic time axis, for radial and non-radial samples, measured from immediately after unloading to 6 months post-compression. Shaded bands show standard deviation across three samples (n=3).

Figure 8 presents the recovery of the height of all cork samples after loading–unloading. A logarithmic scale is used in the time axis due to the wide range of time intervals covered by the measurements.

The data presented in Figure 8 indicate that recovery is fastest shortly after unloading. With most of the deformation recovering after the first two hours in both directions. Later on, the rate of recovery decreases significantly and it continues to recover at a much slower rate over the following two weeks. Between the period of time of about two weeks and six months after unloading, the data show an increase in recovery once again, approaching the rate achieved immediately after unloading. Considering the logarithmic time axis, these transitions should not be interpreted as distinct stages or regimes, but rather that the time dependence of recovery does not follow a single logarithmic linear trend, indicating a nonlinear viscoelastic behavior rather than a single mechanism relaxation process.

The non-radial direction consistently exhibited higher recovery than the radial direction at every time point examined through the six-month monitoring period. Throughout the entire recovery period, the difference in recovery between the two directions remains nearly the same; in other words, the viscous behavior tends to be isotropic.

By the end of the observation period, neither radial nor non-radial samples achieved a recovery of 100% by the end of the six-month observation period. The radial samples reached approximately 88% of recovery, while the non-radial samples reached around 92% of their initial height. Therefore, a possible conclusion is that there is a plastic (irreversible) deformation. Yet the visualization in Figure 8 also shows that the recovery is still ongoing. Therefore, we have used older test samples to understand if there is a chance of full recovery after even a longer time period. Additionally, this is supported by other unpublished data that stem from monotonic compression tests, as the samples were found to be fully recovered at the time of measurement performed approximately 20 months after unloading, although the exact time and the behavior after 6 month has not been tracked. In short, we report that a sufficiently long time is needed to characterize the material by a viscoelastic constitutive equation instead of a viscoplastic one.

### 3.3. Material Modeling and Inverse Analysis

In hyperelastic materials, such as in vulcanized rubber, the isochoric approximation is used; however, we have a zero Poisson’s ratio herein such that a non-standard method is followed. Dissipative potential takes into account the time- and history-dependent behavior of the material, including the Mullins-type damage model, which enables us to describe the progressive softening. However, we stress that the material is fully recoverable. This pseudo-elastic damage modeling is understood as the microbuckling or microfolding that is snapping back after releasing the loading. Yet, such mechanisms for recovery cause a large enough delay (viscous behavior) that characteristic times are measured in days, weeks, or even months. Such observations have been reported in the literature [48,49,50]. We present the simplest yet accurate model for lower stress levels in the following: anisotropic hyperelastic, nonlinear viscoelastic, with pseudo-elastic damage softening.

We use continuum mechanics notation and describe the deformation in a material frame. Consider a continuum body, B, with particles, X∈B, denoting material points in the reference configuration. Under a loading, they displace to x. The deformation is measured by the deformation gradient:

(4) $$F=\frac{\partial x}{\partial X},\;J=\det\left(F\right)>0\;.$$

The kinematic response is governed by the right Cauchy–Green deformation tensor $C=F^{T}\cdot F$, with its invariants,

(5) $$\begin{array}{cc} I_{1} & =C:I=\mathrm{tr}(C) \end{array}$$

(6) $$\begin{array}{cc} I_{2} & =\frac{1}{2}\left({\left(\mathrm{tr}\left(C\right)\right)}^{2}-\mathrm{tr}\left(C^{2}\right)\right) \end{array}$$

(7) $$\begin{array}{cc} I_{3} & =\det\left(C\right)=J^{2}\;, \end{array}$$

and regarding the Cayley–Hamilton approach and for an isotropic hyperelastic material, we model a strain energy density, $W=W(I_{1},I_{2},I_{3})$, via the invariants and use Cattaneo’s relation to get the Piola stress tensor, $P_{ij}=\partial W/\partial F_{ji}$. With the usual chain rule,

(8) $$\begin{array}{cc} \frac{\partial W}{\partial C} & =\frac{\partial W}{\partial I_{1}}\frac{\partial I_{1}}{\partial C}+\frac{\partial W}{\partial I_{2}}\frac{\partial I_{2}}{\partial C}+\frac{\partial W}{\partial I_{3}}\frac{\partial I_{3}}{\partial C}\;,\\ \frac{\partial I_{1}}{\partial C} & =I\;,\\ \frac{\partial I_{2}}{\partial C} & =\frac{1}{2}\left(2\;\mathrm{tr}\left(C\right)\frac{\partial \mathrm{tr}(C)}{\partial C}-\frac{\partial \mathrm{tr}(C^{2})}{\partial C}\right)=I_{1}I-C\;,\\ \frac{\partial I_{3}}{\partial C} & =\det\left(C\right)C^{-T}\;, \end{array}$$

and we obtain

(9) $$\begin{array}{c} P_{lk}=\frac{\partial W}{\partial C_{ij}}\frac{\partial C_{ij}}{\partial F_{kl}}=\left(\left(\frac{\partial W}{\partial I_{1}}+\frac{\partial W}{\partial I_{2}}I_{1}\right)\delta_{ij}-\frac{\partial W}{\partial I_{2}}C_{ij}+\frac{\partial W}{\partial I_{3}}I_{3}{\left(C^{-1}\right)}_{ij}\right)\left(\delta_{il}F_{kj}+\delta_{jl}F_{ki}\right)\;. \end{array}$$

This engineering stress measure is then transformed to the true or Cauchy stress

(10) $$\begin{array}{c} \sigma_{mk}=\frac{1}{J}F_{ml}P_{lk}=\frac{1}{J}\left(F_{mi}F_{kj}+F_{mj}F_{ki}\right)\left(\left(\frac{\partial W}{\partial I_{1}}+\frac{\partial W}{\partial I_{2}}I_{1}\right)\delta_{ij}-\frac{\partial W}{\partial I_{2}}C_{ij}+\frac{\partial W}{\partial I_{3}}I_{3}{\left(C^{-1}\right)}_{ij}\right)\;. \end{array}$$

The investigated cork material is transversely isotropic with a radial direction, a, along the cells. We use a unit length for this directional vector, |a|=1. Then the structural tensor, M=a⊗a, becomes normalized and generates two more invariants [51] as follows:

(11) $$\begin{array}{cc} I_{4} & =\mathrm{tr}(C\cdot M)\;,\\ I_{5} & =\frac{1}{2}\left({\left(\mathrm{tr}\left(C\cdot M\right)\right)}^{2}-\mathrm{tr}\left({\left(C\cdot M\right)}^{2}\right)\right)\;. \end{array}$$

We skip $I_{6}=\det\left(C\cdot M\right)$ since det(M)=1, thus, $I_{6}=I_{3}$. The cork material possesses microstructural cellular walls that fold reversibly without lateral expansion, hence, Poisson’s ratio (transversal to the radial deformation) becomes zero. Under uniaxial compression along direction $x_{1}$, with λ=1+ε as the radial stretch, the deformation reads

(12) $$F_{ij}=\left(\begin{array}{ccc} \lambda & 0 & 0\\ 0 & 1 & 0\\ 0 & 0 & 1 \end{array}\right)\Rightarrow C_{ij}=\left(\begin{array}{ccc} \lambda^{2} & 0 & 0\\ 0 & 1 & 0\\ 0 & 0 & 1 \end{array}\right)\;,\;C_{ij}M_{jk}=\left(\begin{array}{ccc} \lambda^{2}a_{1}a_{1} & 0 & 0\\ 0 & a_{2}a_{2} & 0\\ 0 & 0 & a_{3}a_{3} \end{array}\right)\;.$$

Since the compression results in ε<0, stretch is positive but less than one, λ≤1. Therefore, the strain energy density function reduces significantly and we obtain

(13) $$\begin{array}{cc} I_{1} & =\lambda^{2}+2\;,\\ I_{2} & =\frac{1}{2}\left({\left(\lambda^{2}+2\right)}^{2}-\left(\lambda^{4}+2\right)\right)=\lambda^{2}+1\;,\\ I_{3} & =\lambda^{2}\;,\\ I_{4} & =\lambda^{2}a_{1}^{2}+a_{2}^{2}+a_{3}^{2}\;,\\ I_{5} & =\lambda^{2}a_{1}^{2}a_{2}^{2}+\lambda^{2}a_{1}^{2}a_{3}^{2}+a_{2}^{2}a_{3}^{2}\;. \end{array}$$

Obviously, the first three invariants are linearly dependent to each other and we may use only *W* depending on $I_{1}-3$ such that it goes to zero for the reference (initial) condition, λ=1. Also, we use the coordinate system along the radial direction, $a_{i}=\left\{1,0,0\right\}$ with $I_{4}=\lambda_{r}^{2}$, and along the non-radial direction (Tangential), $a_{i}=\left\{0,1,0\right\}$ with $I_{4}=1$. Therefore, the model is a Yeoh-type model with a quartic formulation,

(14) $$\begin{array}{cc} W= & \frac{1}{2}C_{1}{\left(I_{1}-3\right)}^{2}+\frac{1}{3}C_{2}{\left(I_{1}-3\right)}^{3}+\frac{1}{4}C_{3}{\left(I_{1}-3\right)}^{4}+\frac{1}{2}C_{4}{\left(I_{4}-1\right)}^{2}\\ & +\frac{1}{3}C_{5}{\left(I_{4}-1\right)}^{3}+\frac{1}{4}C_{6}{\left(I_{4}-1\right)}^{4}\;, \end{array}$$

where $C_{4}$, $C_{5}$, $C_{6}$ are correction terms to $C_{1}$, $C_{2}$, $C_{3}$, respectively, depending on the loading direction through the structural tensor. For the sake of clarity, we expand for the case of loading in the radial direction,

(15) $$\begin{array}{c} W_{r}=\frac{1}{2}\left(C_{1}+C_{4}\right){\left(\lambda_{r}^{2}-1\right)}^{2}+\frac{1}{3}\left(C_{2}+C_{5}\right){\left(\lambda_{r}^{2}-1\right)}^{3}+\frac{1}{4}\left(C_{3}+C_{6}\right){\left(\lambda_{r}^{2}-1\right)}^{4} \end{array}$$

and for the case of a loading in a non-radial direction,

(16) $$\begin{array}{c} W_{\mathrm{nr}}=\frac{1}{2}C_{1}{\left(\lambda_{\mathrm{nr}}^{2}-1\right)}^{2}+\frac{1}{3}C_{2}{\left(\lambda_{\mathrm{nr}}^{2}-1\right)}^{3}+\frac{1}{4}C_{3}{\left(\lambda_{\mathrm{nr}}^{2}-1\right)}^{4}\;. \end{array}$$

We emphasize that two independent measurements are needed; one for obtaining $C_{4}$, $C_{5}$, $C_{6}$, which are then used for the other one to determine $C_{1}$, $C_{2}$, $C_{3}$. Then the formulation holds for any directional loading since the energy has the mathematical structure needed for a well-conditioned formulation. In this constellation, the Cauchy stress (normal component, $\sigma_{11}$) from Equation (10) becomes

(17) $$\begin{array}{cc} \sigma_{0}^{r}\;=\; & 2\lambda_{r}\left(\left(C_{1}+C_{4}\right)\left(\lambda_{r}^{2}-1\right)+\left(C_{2}+C_{5}\right){\left(\lambda_{r}^{2}-1\right)}^{2}+\left(C_{3}+C_{6}\right){\left(\lambda_{r}^{2}-1\right)}^{3}\right)\;,\\ \sigma_{0}^{\mathrm{nr}}\;=\; & 2\lambda_{\mathrm{nr}}\left(C_{4}\left(\lambda_{\mathrm{nr}}^{2}-1\right)+C_{5}{\left(\lambda_{\mathrm{nr}}^{2}-1\right)}^{2}+C_{6}{\left(\lambda_{\mathrm{nr}}^{2}-1\right)}^{3}\right)\;, \end{array}$$

where we may relate $C_{1/4}>0$ with an initial tangent modulus, while $C_{2/5}$ and $C_{3/6}$ capture cell wall bending, collapse, and the densification type of nonlinearities. In order to account for stress softening during cyclic loading, the so-called Mullins effect needs to be considered. We stress that this effect is a local deformation mechanism in terms of microstructural folding or buckling—we stress that this behavior is sometimes called damage; however, the response is recoverable. In other words, we adopt a pseudo-elastic formulation [52] where the strain energy density, *W*, is modulated by a continuous scalar variable η∈(0,1]. Therefore, energy is defined as

(18) $$\begin{array}{c} \tilde{W}=\eta W \end{array}$$

where *W* is given in Equation (14). Algorithmically, it is simpler to obtain the energy from the stress by using the Cattaneo relation

(19) $$\begin{array}{c} \sigma_{0}\left(\lambda \right)=\frac{\partial W}{\partial \lambda } \end{array}$$

thus the energy is accumulated from the reference state, λ=1, to the current state, as follows:

(20) $$\begin{array}{cc} W_{r} & =\int_{1}^{\lambda_{r}}\sigma_{0}^{r}\;d\hat{\lambda_{r}}\;,\\ {\tilde{W}}_{\mathrm{nr}} & =\eta \int_{1}^{\lambda_{\mathrm{nr}}}\sigma_{0}^{\mathrm{nr}}\;d\hat{\lambda_{\mathrm{nr}}}\;. \end{array}$$

In order to capture the material memory caused by the microfolding, the maximum strain energy density achieved across the load history up to time *t* is recorded as follows:

(21) $$\begin{array}{c} W_{0}^{\max}\left(t\right)=\max_{\tau \leq t}W_{0}\left(\tau \right) \end{array}$$

The damage evolution variable η is governed by the difference between the historical maximum energy state $W_{0}^{\max}$ and the current strain energy $W_{0}$:

(22) $$\begin{array}{c} \eta \left(t\right)=1-\frac{1}{r_{\mathrm{Mul}}}\mathrm{erf}\left(\frac{W_{0}^{\max}-W_{0}}{m_{\mathrm{Mul}}+\beta_{\mathrm{Mul}}\;W_{0}^{\max}}\right) \end{array}$$

where $r_{\mathrm{Mul}}>1$ is a damage saturation ratio, $m_{\mathrm{Mul}}\geq 0$ sets a damage initiation threshold, and $\beta_{\mathrm{Mul}}\geq 0$ steers the rate of damage accumulation at higher stretch levels. They decay by the standard error function

(23) $$\begin{array}{c} \mathrm{erf}\left(x\right)=\frac{2}{\sqrt{\pi }}\int_{0}^{x}\exp\left(-u^{2}\right)du \end{array}$$

leading to a hereditary type of a memory of damage accumulation. The modified (damaged) elastic stress component $\sigma_{e}\left(t\right)$ is computed by scaling the primary hyperelastic stress by the current damage parameter η(t), as follows:

(24) $$\begin{array}{c} \sigma_{e}=\eta \left(t\right)\sigma_{0}\left(t\right)\;. \end{array}$$

This formulation produces two distinct behaviors depending on loading and unloading cases. While loading, $W_{0}=W_{0}^{\max}$, the argument of the error function reads zero, practically erf(0)=0⇒η=1. The response follows the undamaged curve $\sigma_{e}=\sigma_{0}$. While unloading or after unloading in the case of reloading, $W_{0}<W_{0}^{\max}$, the damage variable degrades to η<1, causing stress softening below the primary envelope. Once the reloading path reaches $W_{0}^{\max}$, then η returns to 1. Standard models in rheology, such as the Maxwell or Kelvin–Voigt elements, assume a constant viscosity, $\mu_{0}$. The implementation of one and many chains shows an inaccurate representation of the experimental results. We also emphasize the discussion regarding Figure 8, where the nonlinear viscous characterization has been suggested. Also, the literature indicates that for cellular materials there is a rate dependency during dynamic compression [53,54]. Therefore, we extend classical linear viscoelasticity by introducing a strain-rate-dependent effective viscosity, $\mu_{\mathrm{eff}}\left(\dot{\varepsilon }\right)=g\left(\dot{\varepsilon }\right)\mu_{0,\mathrm{vis}}$, by means of a Johnson–Cook type of modeling:

(25) $$\begin{array}{c} g\left(\dot{\varepsilon }\right)=1+C\ln\left(1+{\left(\dot{\varepsilon }\right)}^{2}\right)\;. \end{array}$$

We decompose stress into elastic and viscous part, $\sigma =\sigma_{e}+\sigma_{v}$. For the viscous part, we use

(26) $$\begin{array}{c} {\dot{\sigma }}_{v}+\frac{1}{\tau_{\mathrm{vis}}}\sigma_{v}=\mu_{\mathrm{eff}}\;\dot{\varepsilon }\;. \end{array}$$

For solving the latter equation within time steps, $\Delta t_{i}=t_{i}-t_{i-1}$, we use an integrating factor method assuming piecewise-constant strain rate over the interval $\left[t_{i-1},t_{i}\right]$. By simply rewriting as

(27) $$\begin{array}{c} \frac{d}{dt}\;\left(\sigma_{v}\exp\;\left(\frac{t}{\tau_{\mathrm{vis}}}\right)\right)=\mu_{\mathrm{eff}}\;\dot{\varepsilon }\exp\left(\frac{t}{\tau_{\mathrm{vis}}}\right)\;, \end{array}$$

we may now integrate over the time step $\left[t_{i-1},t_{i}\right]$, written as a list in time, $\sigma_{v}\left(t_{i}\right)=\sigma_{v}^{i}$, and similarly,

(28) $$\begin{array}{c} \sigma_{v}^{i}\;\exp\left(\frac{t_{i}}{\tau_{\mathrm{vis}}}\right)-\sigma_{v}^{i-1}\;\exp\left(\frac{t_{i-1}}{\tau_{\mathrm{vis}}}\right)=\mu_{\mathrm{eff}}^{i-1}\;{\left(\dot{\varepsilon }\right)}^{i-1}\tau_{\mathrm{vis}}\left(\exp\left(\frac{t_{i}}{\tau_{\mathrm{vis}}}\right)-\exp\left(\frac{t_{i-1}}{\tau_{\mathrm{vis}}}\right)\right)\;. \end{array}$$

By multiplying the latter by $\exp(-t_{i}/\tau_{\mathrm{vis}})$, we obtain

(29) $$\begin{array}{c} \sigma_{v}^{i}=\sigma_{v}^{i-1}\exp\left(\frac{-\Delta t_{i}}{\tau_{\mathrm{vis}}}\right)+\mu_{\mathrm{eff}}^{i-1}{\left(\dot{\varepsilon }\right)}^{i-1}\left(1-\exp\left(\frac{-\Delta t_{i}}{\tau_{\mathrm{vis}}}\right)\right)\;. \end{array}$$

The implementation of such a model is its strength. Even more advanced models are available using fractional derivatives for such long characteristic times [55], yet their implementation in an inverse analysis becomes more challenging.

Experimental data are acquired at a high frequency and may contain measurement noise such that the data are smoothed using the Savitzky–Golay filter from SciPy (stable version 1.18),

(30) $$\begin{array}{c} {\hat{y}}_{i}=\sum_{j=-M}^{M}c_{j}y_{i+j}\;. \end{array}$$

A window size of 91 points and polynomial order 2 are employed to extract derivative values ${\dot{\varepsilon }}_{i}$. The inverse problem identifies the following parameters:

(31) $$\begin{array}{c} \theta =\{C_{1},C_{2},C_{3},C_{4},C_{5},C_{6},r_{\mathrm{Mul}},m_{\mathrm{Mul}},\beta_{\mathrm{Mul}},\mu_{0,\mathrm{vis}},C_{\mathrm{vis}},\tau_{\mathrm{vis}}\}\;, \end{array}$$

by minimizing the mean squared error; in other words, the objective function becomes

(32) $$\begin{array}{c} J\left(\theta \right)=\frac{1}{N}\sum_{k=1}^{N}{\left(\sigma \left(t_{k};\theta \right)-\sigma_{\exp}\left(t_{k}\right)\right)}^{2} \end{array}$$

subject to physical inequality constraints (lower and upper bounds) $\theta_{lb}\leq \theta \leq \theta_{ub}$. Parameter identification uses the quasi-Newton L-BFGS-B algorithm to get the arguments minimizing the objective function,

(33) $$\begin{array}{c} \theta^{*}=\arg\min_{\theta }J\left(\theta \right)\;. \end{array}$$

**Figure 9:**
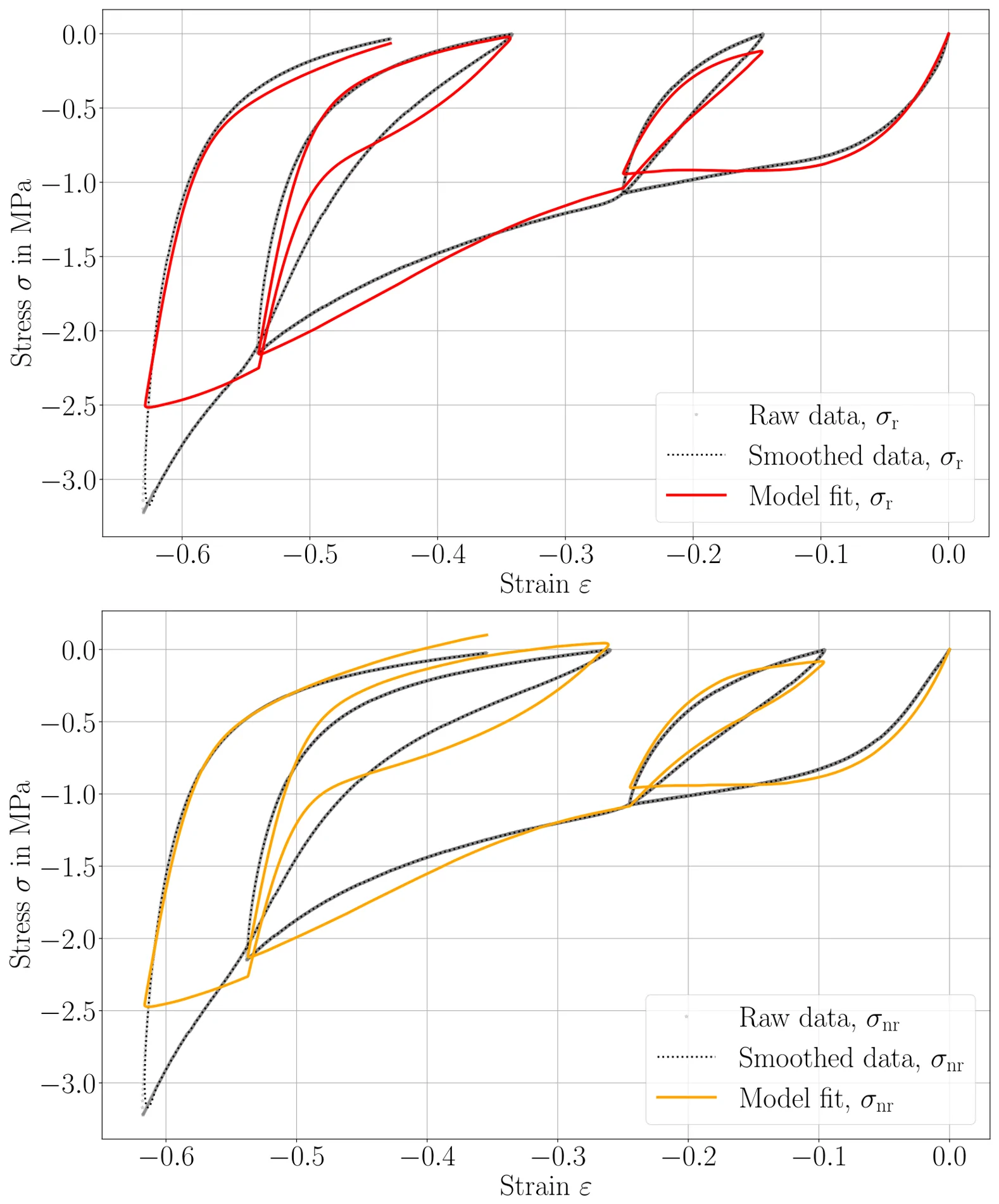
Inverse analysis results, (**top**): in the radial direction; (**bottom**): in the non-radial direction. Raw data and their smoot curve are shown in black and the fit curve in red (**top**) and orange (**bottom**).

This nonlinear regression problem is solved by using the SciPy methods by implementing both elastic tangent stiffness and hereditary integral in a single vector loop for high efficiency. Since we solve a nonlinear regression problem, there exist several solutions. Therefore, the initial parameters may be important to obtain one or another solution. We have performed 50 random runs for different initial parameters; however, this inverse problem is large in the convex part, so for a sizable interval of parameters, the algorithm has converged to the same solution. The optimized material parameters obtained from quasi-static cyclic compression test data are summarized in Table 3, which demonstrates agreement across loading, unloading, and reloading regimes in Figure 9.

## 4. Discussion

In all levels of stress, the radial specimen reaches a specific value of stress at a lower value of strain than the non-radial specimen, which follows the logic of compressive loading along the direction of the cork cells. This stiffer behavior in the radial direction has also been observed in softwood in the direction of the tracheid orientation [56], which can be qualitatively explained by the different buckling patterns in honeycomb structures along the cells vs. transverse to the cells [15]. This feature remains almost the same throughout the stress range but diminishes to some extent at the higher stress parameters (10 and 15 MPa), indicating that with the denser material, the advantage in terms of compliance of the non-radial specimen is partially lost. Densification behavior in cork appears to be caused by the cellular essence of the material and not the direction of the compression.

This densification behavior is also evident in the shape of the loading curves, where radial direction response rises steeply toward each target stress while non-radial direction response has a flatter shape, growing over the whole loading range. This observation signifies the stiffening response that is softer and more progressive, instead of being focused only on larger stresses. A possible explanation may be that microbuckling mechanisms are anisotropic. Yet the nonlinear viscous behavior makes it challenging to verify this assumption. Therefore, in the inverse analysis, we have tried different $\left\{r_{\mathrm{Mul}},m_{\mathrm{Mul}},\beta_{\mathrm{Mul}}\right\}$ values for radial and non-radial specimens. The accuracy failed to increase. Therefore, we understand that the microbuckling causing pseudo-elastic damage is an anisotropic phenomenon.

We highlight herein the analysis of repeatability in Table 2. The spread of the peak stress in both directions is relatively small. Since the tests were displacement-controlled (the tests were conducted under constant crosshead speed without controlling the stress and only measuring it), the cycle was finished once the stress reached the required value (for instance, 1, 2, 3, 4, 5, 10, or 15 MPa). The small stress spread, therefore, indicates the accuracy in reaching the stopping criterion according to the different tests, rather than direct stress control. In contrast, the strain ratio is at the maximum at low stress in both directions. This result arises due to the low absolute strain values observed at low stress, which tend to magnify insignificant differences in the sample due to the lack of complete or even initial contact of the sample with the loading plates prior to sufficient contact being achieved. With the increase in stress, the relative strain spread becomes smaller since the material enters the collapse and densification regimes, which leads to a more uniform behavior in all samples. At the same time, we used different loading rates that the nonlinear viscous material behavior may mask to identify the dominating mechanism, if it is softening- or viscosity-related.

There is evidence of clear softening in the two directions during consecutive cycles, which is indicative of the Mullins-type behavior in which each reloading track shows a value below the extrapolated value from the previous cycle. This behavior agrees with direct observations of microstructure presented in the literature, as it has been shown in scanning electron images of cork cell structure after compression that the walls fold and sway under load without cracking, including during densification [34]. These observations imply that the identified stress-softening should be considered a recoverable and pseudo-elastic failure mechanism due to the local deformation of the cell walls and not permanent damage. Both directions showed a residual strain that increases with the stress, but it was more pronounced in the radial direction. This may imply that the buckled cell walls in this direction are not able to recover quickly enough within the time of the experiment while the recovery was quick in the other direction due to the distributed, progressive character of the buckling.

The loading–unloading pattern signifies the interplay of softening and pseudo-elastic damage softening. Radial and non-radial cuts from the same branch present the anisotropic nature of the cork. Herein we stress that the non-radial response was measured in the tangential direction only such that we implemented a transversely isotropic response. We emphasize that the recovery takes time, yet we understand that the hyperelastic-viscoelastic-softening behavior is fully recoverable (see Appendix A). The first three cycles have been successfully fitted by this advanced model that is still the simplest. The results in Figure 9 show a relatively good fit in the lower stress levels up to 50% strain, the first two cycles. We emphasize that the loading rate is different in the first and second cycles. The hysteresis characteristics are also slightly different in the experiment, yet the model has a more distinguished difference in this part. Since the nonlinear viscous part is strain-rate-dependent, a possible explanation is that the Mullins type softening may need an extension for strain rate dependency. Such an outcome is only visible by a cyclic test with varying strain rate. Relaxation tests assume zero strain rate and creep tests run at a varying strain rate for accommodating zero stress. Therefore, such an analysis from relaxation tests, leading to the form of the constitutive model herein, may be challenging.

Owing to the nonlinear material response and difficulties in inverse analysis, one-dimensional counterparts are used in the literature. Even if their accuracy is high in fitting the measurements, their implementation in engineering applications may be limited since a one-dimensional formulation cannot be used in a three-dimensional numerical implementation, see Section 1.4 in [57] for further discussions. Therefore, we suggest starting with a constitutive model by using a three-dimensional formulation. In this way, the material parameters may be directly used in finite-element-method-based solid mechanics computations. However, the presented formulation has only been developed based on uniaxial compression experiments and is therefore valid only for the compressive behavior of cork. Under tensile loading, the behavior may change due to the cellular structure [58,59]. This distinction is often discarded in elasticity; yet the tensile/compression asymmetry is a known phenomenon and its implementation necessitates generalized elasticity [60]. Therefore, we model the behavior as usual in elasticity by using the first-gradient models and refrain from recommending using the model under tensile or mixed loading scenarios.

Testing was performed only for radial and tangential directions. No transversal [4,8,18,34] compression tests were conducted, and the authors relied on the existing literature for the equivalence of axial and tangential responses. Testing the transversal direction should be a useful extension of this work.

## 5. Conclusions

The underlying work describes a constitutive model for cork that involves anisotropy and is capable of explaining how the cork reacts to repeated compression. The solution combines hyperelastic and viscoelastic modeling with Mullins-type stress softening. The three-dimensional formulation improves the ability to gain a further understanding of cork’s behavior, which is important for computer simulations of practical applications like sealing, cushioning, and energy absorption. By including the directional differences and the effect of progressive softening, this method is said to enhance the ability to predict the behavior of cork under repeated loading.

Some limitations must be taken into account. The investigation carried out only studied the radial and tangent directions under the assumption that the mechanical behavior is similar in the tangential and transverse directions. The monitoring of long-term recovery was carried out for six months, which is not enough to determine the time for the full recovery. We note that after 20 months we have measured the full recovery such that the material shows no inelasticity but a very long relaxation time. In subsequent studies, a longer duration of recovery measurement should be used together with the data of creep and stress relaxation to quantify the time-dependent behavior. More detailed studies may focus on the tensile properties of the material and working parameters such as temperature and humidity so that the model is tested with a broader service condition range.

## Figures and Tables

**Table 1:** Loading–unloading rate applied until the given stress level of this so-called stepped compression protocol. Rates were used until the target stress was obtained. This protocol measured the influence of the strain rate and stress magnitude on the material response.

Cycle nr	Target Stress (MPa)	Loading/Unloading Rate (mm/min)
1	1	1
2	2	2
3	3	3
4	4	4
5	5	5
6	10	1
7	15	1

**Table 2:** Cycle-by-cycle repeatability of the loading–unloading tests, expressed as the relative spread $\Delta X/\bar{X}=\left(X_{\max}-X_{\min}\right)/\bar{X}$ in peak stress and peak strain across three repeated tests, for the radial (r) and non-radial (nr) directions. Peak values are reported as $\bar{X}\pm \frac{1}{2}\Delta X$.

Target Stress (MPa)	$\Delta \sigma_{r}/{\bar{\sigma }}_{r}$ (%)	$\Delta \varepsilon_{r}/{\bar{\varepsilon }}_{r}$ (%)	$\Delta \sigma_{\mathrm{nr}}/{\bar{\sigma }}_{\mathrm{nr}}$ (%)	$\Delta \varepsilon_{\mathrm{nr}}/{\bar{\varepsilon }}_{\mathrm{nr}}$ (%)
1	0.34	69.93	0.26	92.06
2	0.11	9.96	0.23	16.83
3	0.08	9.12	0.41	9.60
4	0.05	8.77	1.83	6.71
5	0.43	8.58	2.70	5.21
10	0.07	8.06	0.13	3.24
15	0.10	7.87	0.27	2.27

**Table 3:** Optimized constitutive model parameters and variable definitions.

	Symbol	Name	Value	Units
Hyperelastic	$C_{1}$	Elastic modulus	0.026894	MPa
	$C_{2}$	Elastic modulus	0.333034	MPa
	$C_{3}$	Elastic modulus	0.423475	MPa
	$C_{4}$	Elastic modulus	3.867187	MPa
	$C_{5}$	Elastic modulus	11.420617	MPa
	$C_{6}$	Elastic modulus	12.321750	MPa
Damage	$r_{\mathrm{Mul}}$	Damage saturation ratio	1.480442	−
	$m_{\mathrm{Mul}}$	Damage initiation threshold	0.053015	${\mathrm{MJ}/m}^{3}$
	$\beta_{\mathrm{Mul}}$	Energy dependence coefficient	0.037555	−
Viscoelastic	$\mu_{0,\mathrm{vis}}$	Viscosity	199.025747	MPa·s
	$C_{\mathrm{vis}}$	Johnson–Cook rate parameter	0.510487	−
	$\tau_{\mathrm{vis}}$	Relaxation time	34.531013	s

## Data Availability

The original contributions presented in this study are included in the article. Further inquiries can be directed to the corresponding author.

## Appendix A. Recovery Data

Table A1 presents the full dimensional recovery dataset for the radial and non-radial directions, corresponding to the trends shown in Figure 7.

**Table A1:** Percentage recovery of specimen height over time following loading–unloading testing.

Time	Radial (%)	Non-Radial (%)
Right after	68.94	72.46
30 min	74.72	78.91
1 h	76.61	81.12
2 h	78.47	81.95
24 h	79.28	83.88
48 h	79.96	84.56
72 h	80.18	84.89
1 week	81.09	85.93
2 weeks	81.74	86.71
3 months	85.63	90.24
6 months	87.84	91.76
